# Effects of Modified Glucosamine on the Chondrogenic Potential of Circulating Stem Cells under Experimental Inflammation

**DOI:** 10.3390/ijms241210397

**Published:** 2023-06-20

**Authors:** Marco Gasparella, Carola Cenzi, Monica Piccione, Valentina Noemi Madia, Roberto Di Santo, Valeria Tudino, Marco Artico, Samanta Taurone, Chiara De Ponte, Roberta Costi, Rosa Di Liddo

**Affiliations:** 1 Local Health Unit Treviso, Department of Pediatric Surgery, 31100 Treviso, Italy; 2 Department of Pharmaceutical and Pharmacological Sciences, University of Padova, 35131 Padova, Italy; 3 Department of Drug Chemistry and Technology, University of Rome “La Sapienza”, 00185 Rome, Italy; 4 Department of Sensory Organs, University of Rome “La Sapienza”, 00185 Rome, Italy; 5 Department of Movement, Human and Health Sciences-Division of Health Sciences, University of Rome “Foro Italico”, 00185 Rome, Italy

**Keywords:** glucosamine, TNFα, TNF receptors, cartilage, inflammation, CD73, IκBα

## Abstract

Glucosamine (GlcN) is a glycosaminoglycan (GAGs) constituent in connective tissues. It is naturally produced by our body or consumed from diets. In the last decade, in vitro and in vivo trials have demonstrated that the administration of GlcN or its derivates has a protective effect on cartilage when the balance between catabolic and anabolic processes is disrupted and cells are no longer able to fully compensate for the loss of collagen and proteoglycans. To date, these benefits are still controversial because the mechanism of action of GlcN is not yet well clarified. In this study, we have characterized the biological activities of an amino acid (AA) derivate of GlcN, called DCF001, in the growth and chondrogenic induction of circulating multipotent stem cells (CMCs) after priming with tumor necrosis factor-alpha (TNFα), a pleiotropic cytokine commonly expressed in chronic inflammatory joint diseases. In the present work, stem cells were isolated from the human peripheral blood of healthy donors. After priming with TNFα (10 ng/mL) for 3 h, cultures were treated for 24 h with DCF001 (1 μg/mL) dissolved in a proliferative (PM) or chondrogenic (CM) medium. Cell proliferation was analyzed using a Corning^®^ Cell Counter and trypan blue exclusion technique. To evaluate the potentialities of DCF001 in counteracting the inflammatory response to TNFα, we measured the amount of extracellular ATP (eATP) and the expression of adenosine-generating enzymes CD39/CD73, TNFα receptors, and NF-κB inhibitor IκBα using flow cytometry. Finally, total RNA was extracted to perform a gene expression study of some chondrogenic differentiation markers (COL2A1, RUNX2, and MMP13). Our analysis has shed light on the ability of DCF001 to (a) regulate the expression of CD39, CD73, and TNF receptors; (b) modulate eATP under differentiative induction; (c) enhance the inhibitory activity of IκBα, reducing its phosphorylation after TNFα stimulation; and (d) preserve the chondrogenic potentialities of stem cells. Although preliminary, these results suggest that DCF001 could be a valuable supplement for ameliorating the outcome of cartilage repair interventions, enhancing the efficacy of endogenous stem cells under inflammatory stimuli.

## 1. Introduction

Glucosamine (GlcN) (179.17 g/mol) comprises modified glucose with an amine group replacing the hydroxyl group on carbon two [1]. It is distributed throughout the human body (i.e., skin, tendons, ligaments, and cartilage) as an essential component of glycoproteins, proteoglycans, and glycosaminoglycans [2]. It is synthesized via the hexosamine biosynthetic pathway from fructose 6-phosphate and glutamine [3] and then metabolized into GlcNAc for the synthesis of glycosylated proteins and lipids [4,5]. In addition to biochemical functions and significant contributions to tissue structural scaffolding, cell hydration, and cell signaling [6], GlcN and its acetylated derivatives represent one of the most popular over-the-counter (OTC) dietary supplements to prevent or treat a wide variety of inflammatory diseases of the central nervous system [7] and the connective tissues [8,9]. Indeed, Grigorian et al. [7] demonstrated that N-acetylglucosamine inhibits T-helper 1 (Th1) and T-helper 17 (Th17) responses and attenuates the clinical severity of myelin-oligodendrocyte-glycoprotein-induced experimental autoimmune encephalomyelitis (EAE) when administered after disease onset. Moreover, although the efficacy of GlcN in the treatment of chronic cartilage inflammation is still a controversial issue, glucosamine is included in the class of symptomatic slow-acting drugs for osteoarthritis (OA) and is recommended by most European scholarly societies to provide some pain relief in people with osteoarthritis of the knee [9,10,11,12,13]. To date, the common forms of GlcN supplements comprise glucosamine hydrochloride, glucosamine sulfate, and N-acetyl glucosamine [14]. They are usually prepared from chitin via chemical and enzymatic hydrolysis and microbial production [15,16,17,18,19,20,21,22]. Furthermore, the production of these amino sugars from microbial fermentation processes is also performed using fungi or genetically modified bacteria [23]. Pharmacokinetic studies have shown that very large doses of glucosamine (1500 mg/day, ~23 mg/Kg) could be administered orally without evidence of toxicity, but the required therapeutic concentration to inhibit cartilage inflammation is not always reached in plasma and tissue [9,24,25,26,27]. To resolve the stability issues associated with GlcN and GlcNAc, different formulations of this dietary supplement have been developed [25,28,29] to enhance its gut absorption and bioavailability. When administered as a nutraceutical [25], GlcN uptake in cells occurs upon insulin stimulation [30] and via glucose transporters (GLUT1, 2, and 4) [31]. Thus, GlcN is phosphorylated into GlcN-6-phosphate by hexokinases and converted into uridine diphosphate-N-acetylglucosamine (UDP-GlcNAc) via the hexosamine biosynthetic pathway [31]. Thereafter, UDP-GlcNAc is used as a donor substrate by O-linked-N-acetylglucosamine (O-GlcNAc) transferase (OGT), which catalyzes the transfer of O-GlcNAc to a hydroxy group of serine and threonine residues in the target proteins [32]. It is known that O-GlcNAc modification is one of the principal post-transcriptional modifications, and it is observed in several cellular functions, including gene expression, signal transduction, and the subcellular localization of proteins [33]. Under chronic inflammation, the altered form of amino sugars or the depletion of proteoglycans due to the excessive production of matrix metalloproteinases (MMPs) may cause tissue repair defects and pathologies [34,35]. For instance, the lack of proteoglycan precursors has been correlated with the structural and functional defects of skeletal joints [36]. Notably, in the gut mucosa, the alteration of matrix GAGs is frequently observed in bowel diseases, such as ulcers, colitis, chronic proctitis, and Crohn’s disease [37]. Since it is a component of hyaluronic acid, which is largely expressed in cartilage, connective tissue, and synovial fluid, GlcN is estimated to maintain the structure and function of joints [36,37]. In vitro and in vivo settings have demonstrated that GlcN and its derivates exert both anti-inflammatory and chondroprotective effects [7,22,26,38,39,40,41,42]. In particular, they have been shown to suppress the production of inflammatory mediators in vitro (i.e., nitric oxide, prostaglandin E2, and interleukin (IL)-1β and IL-8) in both chondrocytes [43,44,45,46] and synovial cells [47]. Similarly, under in vivo settings, the oral treatment with GlcN alone [5,9,11,26,48,49,50] or in combination with chondroitin sulfate [51,52] or the treatment with glucosamine sulfate combined with etoricoxib [53] was demonstrated to modulate the inflammatory process, decrease collagen degradation by inhibiting the synthesis of MMPs (i.e., MMP13) [39,40], and contextually stimulate the production of cartilage structural components (i.e., collagen II, aggrecan, and sulfated GAG) [38,54], thus limiting the degradation of tissue extracellular matrix. To date, the detailed mechanism by which GlcN downregulates cartilage inflammation is still under evaluation. Several studies suggest that the antioxidant and anti-inflammatory activities of glucosamine are mostly exerted by (i) the modulation of NF-κB by IKKalpha kinase inhibition [26,43] or (ii) the regulation of TNFα signaling [44]. The regulation of the NF-κB inflammatory pathway is mediated by inhibitors belonging to the IκB family, such as IκBα [55]. IκB is an intrinsically unstable protein [56] that exists as two distinct pools in cells: The larger IκB pool binds and retains NF-κB in the cytoplasm at an inactive state, and the minor pool remains as a “free” protein. In resting cells, basal IKK activity phosphorylates bound IκBα and targets it for ubiquitin-dependent degradation. In addition, free IκBα is continuously synthesized and degraded in an IKK- and ubiquitin-independent mechanism, keeping NF-κB from being activated under resting cell conditions. Upon TNFα stimulation, it is rapidly degraded following the phosphorylation mediated by IKKalpha kinase at serine residues 32 and 36, translocates to the nucleus, and binds to target gene promoters, including IκB [57]. Compared to other IκB proteins, IκBα responds most rapidly to stimuli and mediates a powerful negative feedback loop that terminates NF-κB transcriptional activity [55]. Accumulating evidence implicates a pivotal role of the transcription factor NF-κB in the regulation of cell growth and the differentiation of mesenchymal stem cells (MSCs) [58,59,60]. Pro-inflammatory cytokine TNFα is largely expressed during cartilage inflammation and interferes with the healing process of chondral and osteochondral defects, increasing the expression of aggrecanase and decreasing the expression of proteoglycans [61,62]. Studies investigating the biological effects of anti-TNFα drugs demonstrated that the inhibitors of TNFα have substantial chondroprotective activity [63,64] in mesenchymal stem cells and non-stem cell populations. MSCs are known to contribute to the structural and functional restoration of injured tissues by counteracting the inflammatory activity of TNFα [65]. The site-specific administration of MSCs to patients affected by chronic inflammatory diseases or the mobilization of endogenous stem cells to damaged sites are effective anti-inflammatory strategies at disease onset but could fail to alleviate symptoms during disease progression [64]. It has been demonstrated that MSCs are susceptible to environmental changes, and the exposure to TNFα could cause their conversion from an immunosuppressive to pro-inflammatory status [66]. Sitcheran et al. [67] reported that NF-κB activated by TNFα inhibits mesenchymal stem cells to undergo chondrogenesis. Interestingly, glucosamine stimulated MSCs to acquire the chondrogenic phenotype and inhibited extracellular matrix degradation [39], suggesting a negative interaction with the NF-κB pathway.

It is known that TNFα exerts its biological activities via two receptors: TNFR1 and TNFR2 [68]. Stem cells, including CMCs, express both receptors and exert immunosuppressive and reparative activities via cell–cell contact and paracrine mechanisms [66,69]. TNFR1 is expressed ubiquitously, interacts with the membrane (mTNFα) or soluble TNFα (sTNFα), triggers cell apoptosis, or regulates the adaptive immune system, promoting the generation of regulatory T cells (Tregs) or repressing the functions of T effector and B effector cells. Inversely, TNFR2 is expressed in immune cells, endothelial cells, mesenchymal stem cells, and neural cells [66]. It preferentially binds to mTNF and mediates anti-inflammatory effects, activating pro-survival genes via NF-κB, ERK, JNK, and p38 MAPK pathways. In cardiac models, the ablation of the TNFR1 gene blunts injury and improves survival, whereas the ablation of the TNFR2 gene exacerbates tissue injury and reduces cell survival [70]. This has led to the important appreciation that TNFα may have beneficial or detrimental effects depending on which of its receptors is activated. Thus, targeting TNFα signaling could be of great therapeutic value for the development of stem-cell-based therapies for cartilage repair in osteoarthritis and related orthopedic conditions. Chondrocytes and mesenchymal stem cells secrete numerous bioactive molecules, including adenine nucleotides and nucleosides [71]. Native articular cartilage is challenged by synovial fluid flow during normal joint motion, and ATPs released together with adenosine production are transiently increased in the joint microenvironment. Following shockwave trauma or excessive joint motion, cartilage homeostasis is drastically altered, resulting in excessive ecto-5′-nucleotidase/CD73 production, adenosine accumulation, a disproportionate activation of adenosine A2B receptors, and a significant release of pro-inflammatory mediators.

In this study, we assessed an in vitro model of chondrogenesis based on human circulating multipotent cells to evaluate the potentiality of an amino acid (AA) derivate of GlcN, called DCF001, in counteracting the detrimental effects of TNFα on cell viability, ATP release, TNFα receptors, CD73 expression, and the chondrogenic potentialities of human circulating multipotent stem cells cultured under proliferative (PM) or chondrogenic (CM) conditions. The preparation of DCF001 was performed according to Katritzky and colleagues [19,20], which demonstrated that conjugation with AA increases the bioavailability of GlcN and enhances its pharmaceutical activity.

## 2. Results

### 2.1. In Vitro Model

**Figure 1:**
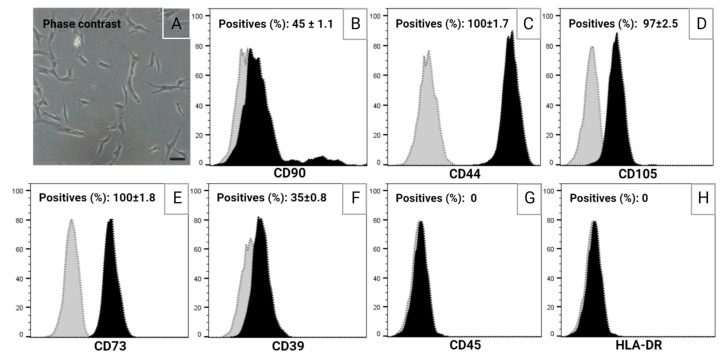
(**A**) Optical microscopy of CMC cells. Bar: 10 µm. (**B**–**H**) Flow cytometry analysis of subconfluent cultures. IgG control (grey peak); target protein (black peak). Data were normalized to the peak height (number of events), resulting in relative percentages (%) ± standard deviation (SD). The analysis was performed using FlowJo™ v10.8.1 Software (Ashland, OR, USA).

The biological effects of DCF001 were investigated in CMCs expanded up to the fifth passage. As shown in Figure 1, cell populations exhibited a typical fibroblast-like morphology and characteristic stem cell markers, including CD90 (45 ± 1.1%), CD44 (100 ± 1.7%), and CD105 (97 ± 2.5%). Furthermore, the expression of enzymes related to ATPase activity, including CD73 (ecto-5′-nucleotidase) (100 ± 1.8%) and CD39 (ecto-ATPase) (35 ± 0.8%), was also demonstrated. As expected, CD45 and HLA-DR, which are typically expressed in mature hematopoietic cells, were not detected.

### 2.2. Cell Viability and ATP Release

**Figure 2:**
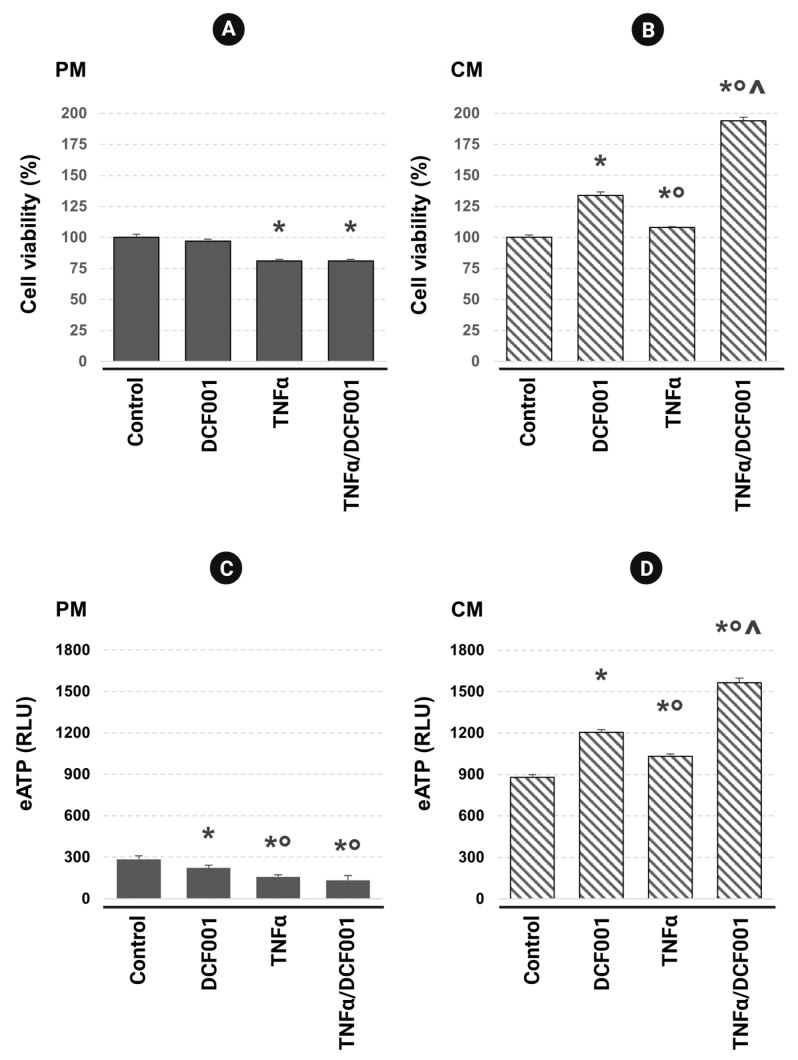
Cell viability analysis on CMCs pretreated with TNFα (10 ng/mL) for 3 h and then stimulated for a further 24 h with DCF001 (1 µg/mL) in a proliferative (**A**) or chondrogenic (**B**) medium. Data were expressed as a percentage (%) ± SD of viable cells detected using cell counting with the trypan blue exclusion of dead cells. In parallel, extracellular ATP (eATP) in PM (**C**) and CM (**D**) cultures was evaluated using the CellTiter-Glo^®^ Luminescent Cell Viability Assay. Luminescence was read with a VICTOR^®^ Nivo™ Plate Reader (PerkinElmer, Waltham, MA, USA) and then reported in relative light units (RLU). * *p*: vs. control; ° *p*: vs. DCF001-induced cells; ^ *p*: vs. TNFα-primed cells.

DCF001 showed its ability to preserve cell growth and the differentiative potential of CMCs under experimental inflammation. As observed in Figure 2A, TNFα decreased cell proliferation in PM cultures (*p* < 0.05), and this response was not affected by DCF001. In contrast, in CM cultures, the administration of DCF001 upregulated (*p* ≤ 0.01) the cell growth rate (Figure 2B), and this mitogenic effect (*p* ≤ 0.01) was stronger in cells pre-treated with TNFα. In parallel, to investigate whether DCF001 alters stem-cell-growth-modulating ATP signaling, we collected the culture medium of all experimental groups and measured the extracellular ATP content (eATP). As shown in Figure 2C, DCF001 and/or TNFα promoted a significant reduction in eATP (*p* < 0.05) under proliferative conditions. In contrast, the samples cultured with chondrogenic factors (Figure 2D) responded to the stimulation with DCF001 or TNFα by accumulating eATP (*p* ≤ 0.05). This effect significantly increased when DCF001 was added after TNFα priming (*p* ≤ 0.05) (Figure 2D).

### 2.3. Effect of DCF001 on CD39 and CD73 Expression

**Figure 3:**
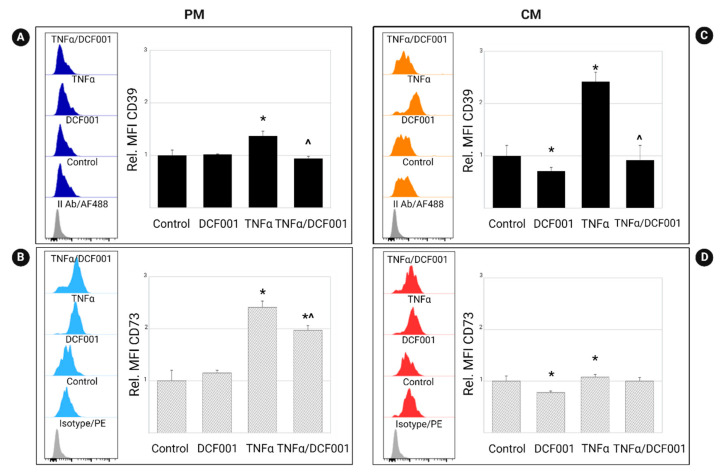
Detection of CD39 (**A**,**C**) and CD73 (**B**,**D**) expression in CMC cells. The samples were pretreated with TNFα (10 μg/mL) for 3 h and thus cultured for a further 24 h with a proliferative (PM) or chondrogenic (CM) medium supplemented with DCF001 (1 µg/mL). Unstimulated cells (control) or samples treated with only DCF001 or TNFα were used as references. The analysis was performed by flow cytometry using direct staining, and data were reported as representative histograms and relative MFI values (Rel. MFI = target MFI/Isotype IgG MFI). * *p*: vs. control; ^ *p*: vs. TNFα-primed cells.

We tested the hypothesis that DCF001 modulates the expression of CD39 and CD73, two ectoenzymes known to convert extracellular ATP to AMP (CD39) and AMP to adenosine (CD73). As reported in Figure 3, in comparison to unstimulated cells, CMCs pre-treated with TNFα and thus cultured for 24 h in a PM medium showed an upregulated expression (*p* < 0.05) of both CD39 (Figure 3A) and CD73 (Figure 3B), as expected under inflammatory conditions. In contrast, when TNFα-primed cells were cultured in a CM medium, we only observed the upregulation (*p* < 0.05) of CD39 (Figure 3C) while CD73 was unchanged or barely increased (Figure 3D). These effects were reversed (*p* ≤ 0.05) by DCF001 in both PM and CM. When added to the chondrogenic medium, DCF001 caused a significant reduction (*p* < 0.05) in the expression of both CD39 and CD73.

### 2.4. Effect of DCF001 on TNFα Receptors

**Figure 4:**
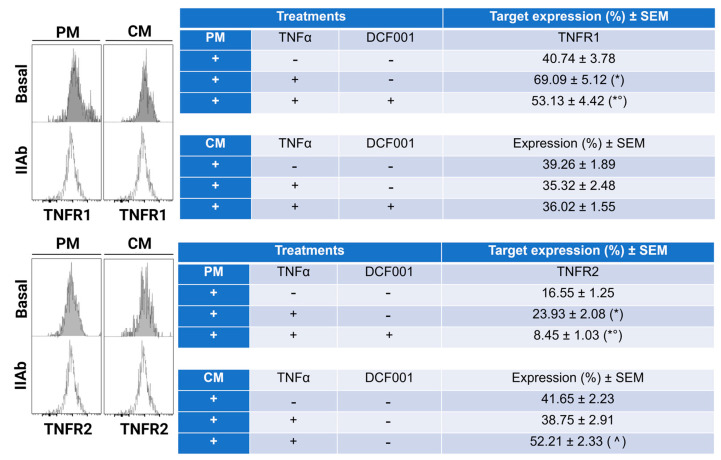
FCM analysis of TNFR1 and TNFR2 in CMC cells cultured in proliferative (PM) or chondrogenic (CM) medium or cells treated with only TNFα (10 μg/mL) and/or DCF001 (1 µg/mL). (*+*) Added to culture medium; (-) not added to culture medium. Histograms represent the basal expression of both TNFR1 and TNFR2 under PM and CM medium. Data from all experimental groups were reported in the table as the mean value of percent positive cells ±SEM. * *p*: vs. control in PM; ° *p*: vs. DCF001-induced cells in PM; ^ *p*: vs. TNFα-induced cells in CM.

We tested the hypothesis that DCF001 exerts protective effects during the chondrogenesis of mesenchymal stem cells by modulating the expression of TNFR1 and TNFR2, which, under inflammatory conditions, mediates pro-inflammatory and anti-inflammatory activities, respectively. As shown in Figure 4, a higher expression of TNFR1 (40.74 ± 3.78%) and TNFR2 (16.55 ± 1.25%) was observed in PM cultures. Following TNFα priming, a significantly (*p* ≤ 0.05) increased expression level of TNFR1 (69.09 ± 5.12%) and TNFR2 (23.93 ± 2.08%) was observed. This response was partially inhibited (*p* ≤ 0.05) by DCF001, as suggested by the reduced expression of both TNFR1 (53.13 ± 4.42%) and TNFR2 (8.45 ± 1.03%). In contrast, under differentiative conditions, the expression of TNFR1 and TNFR2 was not affected by TNFα stimulation. Interestingly, CMCs primed with TNFα and then cultured for 24 h in a CM medium supplemented with DCF001 significantly upregulated (*p* < 0.05) TNFR2 expression (52.21 ± 2.33%) compared to the control (41.65 ± 2.23%) and TNFα-primed cells (38.75 ± 2.91%).

### 2.5. Effect of DCF001 on IκBα

**Figure 5:**
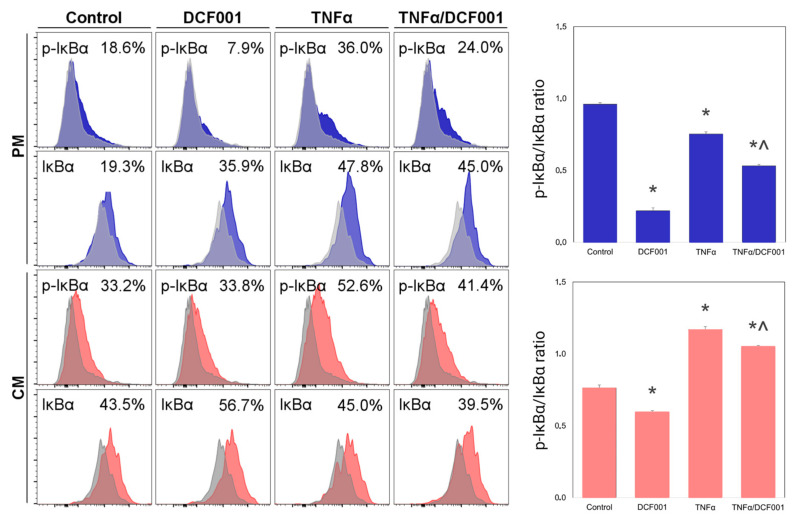
FCM detection of p-IκBα and IκBα in CMC cells cultured for 24 h by regular growth (control) or after treatment with DCF001 and/or TNFα under proliferative (PM) (blue peaks) or chondrogenic (CM) (red peaks) conditions. Data were reported as percentage expression (%) and p-IκB/IκBα expression ratio. The analysis was performed using indirect staining with primary antibodies against mouse anti-human p-IκBα or rabbit anti-human IκBα and Alexa Fluor^®^ 488-conjugated secondary antibodies. Samples treated with only secondary antibodies were used as control (grey peaks). * *p*: vs. control; ^ *p*: vs. TNFα-primed cells.

In resting cells, NF-κB-bound IκBα is subject to slow degradation by both IKK phosphorylation and ubiquitination. In contrast, free IκBα turnover is caused intrinsically by sequences in its C terminus [56]. In the present study, the anti-inflammatory activity of DCF001 was studied by investigating the percentage expression of phosphorylated (p-IκBα) and unphosphorylated IκBα and the p-IκB/IκB ratio. As observed in Figure 5, unstimulated CMC cells showed a low expression of total IκBα and a high (*p* ≤ 0.05) p-IκBα/IκBα ratio (0.96 ± 0.01), thus suggesting a high rate of free protein degradation and NF-κB at an active state. Upon chondrogenic induction, an increased amount of total IκBα but with a significantly decreased (*p* ≤ 0.05) p-IκBα/IκBα ratio (0.76 ± 0.02) was observed and considered indicative of negative regulation of NF-κB. These results were consistent with the literature reporting NF-κB inhibition during the differentiative commitment of MSCs [60]. When CMCs were stimulated with TNFα, p-IκB was upregulated in a chondrogenic medium (p-IκB/IκB ratio: 1.17 ± 0.02, *p* ≤ 0.05) but not in cells kept in a proliferative medium (p-IκB/IκB ratio: 0.75 ± 0.01, *p* ≤ 0.05). Showing a potential inhibitory effect on NF-kB under proliferative (p-IκBα/IκBα ratio:0.22 ± 0.01; *p* ≤ 0.01) and chondrogenic conditions (p-IκBα/IκBα ratio: 0.60 ± 0.01; *p* ≤ 0.05), DCF001 counteracted (*p* ≤ 0.05) the biological effects of TNFα independently of culture conditions (p-IκBα/IκBα ratio: 0.53 ± 0.01 in the PM; p-IκBα/IκBα ratio: 1.05 ± 0.01 in CM).

### 2.6. Gene Expression

**Figure 6:**
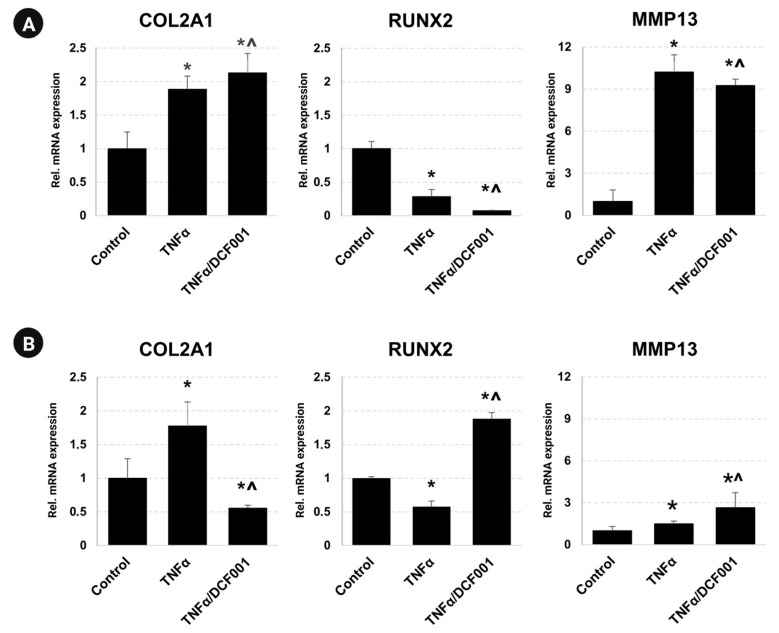
Gene expression study of CMCs stimulated with DCF001 under proliferative (**A**) or chondrogenic (**B**) conditions. Cell cultures were challenged with TNFα (100 ng/mL) for 3 h before stimulation for a further 24 h with DCF001 solubilized in a PM or CM medium. Total RNA was extracted using the TRI Reagent solution and analyzed by quantitative real-time polymerase chain reaction (RT-qPCR). Data were reported as a relative fold increase in gene expression. * *p*: vs. control; ^ *p*: vs. TNFα-primed sample.

As shown in Figure 6, a statistically significant increase in COL2A1 (*p* < 0.05) was observed in PM and CM cultures stimulated with TNFα. In contrast, RUNX2 expression was downregulated (*p* < 0.05). After TNFα priming, DCF001 improved the transcription of COL2A1 (*p* < 0.05) in PM cultures (Figure 6A) while modulating the expression of RUNX2 and MMP13 (*p* < 0.05). In parallel, the cells maintained in the CM medium and DCF001 (Figure 6B) showed increased transcription of RUNX2 and MMP13 (*p* ≤ 0.05). The lower expression of the COL2A1 gene (*p* ≤ 0.05) was observed compared to the references.

## 3. Discussion

Preclinical studies have demonstrated that TNFα release is impaired with respect to symptomatic cartilage defects [72] or inflammation [73,74,75]. Acting as a catabolic factor, it affects chondrocyte viability, promotes the production of metalloproteinases, and inhibits chondrogenic differentiation [61,76]. These biological activities are dose-dependent and involve the inhibition of transcription factor SOX9 via NF-κB signaling [77]. As MSCs express limited proliferation or differentiation when exposed to inflammatory factors [78,79], TNF inhibitors could potentially be beneficial in clinics and could improve the efficacy of the therapeutical treatment of inflammatory diseases [66,80,81,82]. In the present study, we demonstrated that a derivate of glucosamine coupled to tryptophan (DCF001) counteracted the inflammatory effect of TNFα on circulating mesenchymal stem cells (CMCs) via the regulation of cell viability, the accumulation of eATP, and the expression of TNFα receptors and NF-κB inhibitor IκBα. In accordance with the variable effects that TNFα could exert on the fate of MSCs when used alone or combined with differentiation-inducing factors [78], all biological effects of DCF001 were dependent on cell culture conditions after TNFα priming. Li et al. [83] reported that the exposure of MSCs to TNFα during in vitro expansion could be beneficial for cell proliferation, migration, and osteogenic capacity, but these effects are reported to be reversed upon TNFα withdrawal [84]. In contrast, TNFα could exhibit significant inhibitory activity upon stem cell chondrogenesis [62,79] or stimulate a pro-chondrogenic effect when administered before differentiative induction [84]. Our data point towards the role of DCF001 in stem cell differentiation under inflammation that involves extracellular ATP accumulation and the regulation of purinergic receptors CD39/CD73 and TNF receptors [66]. In our study, TNFα priming promoted a significant reduction in cell viability under regular growth conditions while stimulating proliferation under chondrogenic induction. In the absence of differentiation-inducing factors, DCF001 did not affect cell viability nor reversed the inhibitory effect induced by TNFα. In contrast, when administered with chondrogenic factors, glucosamine stimulated the growth of CMCs, with further increased activity in cells primed with TNFα. These data are consistent with the literature reporting that a proliferogenic effect is induced by GlcN in stem cells [39,85] and chondrocytes [39,86]. Because the entry of glucosamine into cells involves glucose transporter systems and this transport is stimulated by insulin, which is a factor added to the chondrogenic medium of CMCs, we hypothesized that DCF001 is more effective when administered with chondrogenic factors because of its increased uptake [24,31]. In our study, the proliferogenic effect of DCF001 correlated with an increase in extracellular ATP, which is a known purinergic controlling mediator of physiological functions [87,88,89,90] via Ca^2+^ signaling [91]. Our data are consistent with studies reporting that, during the chondrogenic maturation of mesenchymal stem cells, the release of ATP occurs in order to shift the cell’s fate from a transient growth phase [92] to chondrogenic differentiation [93]. Kwon et al. [94] demonstrated that ATP is released periodically due to oscillatory secretion during prechondrogenic condensation, and these oscillations are mediated by the P2X4 receptor and intracellular cAMP/PKA pathway. Previously, Iwamoto et al. [93] reported that Pannexin 3 (PANX3), which is a member of the gap junction pannexin family, is expressed in cartilage and functions to shift the chondrocyte’s cell fate from proliferation to differentiation, promoting ATP release into the extracellular space and inhibiting parathyroid hormone (PTH)-mediated cell proliferation, the intracellular levels of cAMP, and the phosphorylation of CREB. Upon inflammatory insults, extracellular ATP could exert pro-inflammatory effects, and MSCs expressing CD39, which catabolizes ATP into AMP, and CD73, which hydrolyzes AMP into adenosine, could transform pro-inflammation into anti-inflammation [95,96], preserving cartilage homeostasis [71]. Based on our results, DCF001 could contribute to chondrogenesis, causing the accumulation of eATP by the desensitization of CD39 and CD73 after activation. Instead, the protective effects of DCF001 against TNFα could involve a modulatory activity on the expression of TNF receptors and the activation of NF-κB. It is known that TNFα affects MSC efficacy in a dose-dependent manner. At higher concentrations, the interaction of TNFα with TNFR1 is known to mediate pro-inflammatory effects via NF-κB activation [97] and reduce MSC efficacy by inhibiting the production of immunosuppressive molecules and growth factors. In contrast to the dual effects of TNFR1, the interaction between TNFα and TNFR2 is effective in only evoking anti-inflammatory effects and cell survival via TNFR-associated factor (TRAF) 1 and 2 proteins [98,99]. While transmembrane TNFα activates both TNFR1 and TNFR2 signaling with high efficacy, soluble TNFα interacts only with TNFR1 in strong and general receptor activation [100]. Compared with wild-type controls, murine BM-MSCs with TNFR2 knockout showed less or no myocardial functional recovery in a rat model of acute ischemia accompanied by the increased production of pro-inflammatory factors [66]. When CMCs were treated with TNFα under proliferative conditions, DCF001 affected the expression of both TNFR1 and TNFR2, suggesting that this GlcN derivate could interfere with the TNFα pathway by lowering the synthesis of its cell surface receptors. These data are in accordance with Scotto et al. [44], who reported a preventive activity exerted by glucosamine on TNFα-induced transcriptional activation in human chondrocytes. Beldi et al. [98] also demonstrated that the TNFα–TNFR2 axis is a crucial regulator of MSC immunological and regenerative functions. In the present study, we demonstrated that DCF001 combined with chondrogenic stimuli promoted an increased expression of TNFR2 in TNFα-primed cells, suggesting that it can stimulate immunosuppressive activity in mesenchymal stem cells. Moreover, due to evidence reporting the involvement of TNFR2 in cell differentiation [61], its increased expression, which is promoted by DCF001, the increase in cell viability and eATP, and the reduced expression of CD39 and CD73 could be indicative of promoted chondrogenesis under inflammation, as previously demonstrated for other molecules, i.e., Atsttrin [99]. The anti-inflammatory effect of DCF001 was further confirmed by the inhibited phosphorylation of IκBα, while its differentiative potential was demonstrated by the expression of chondrogenic gene markers COL2A1, RUNX2, and MMP13 [101].

Different chondrocyte phenotypes are identified during maturation based on their collagen gene expression [102,103]. In the literature, it is reported that the supplementation of the differentiation medium with glucosamine has an improved differentiative effect on stem cells [104]. Chondroprogenitor cells are characterized by the expression of type II collagen (COL2A1) [105,106], while mature chondrocytes express the typical cartilage collagen types II (COL2B), IX, and XI as well as aggrecan [107,108,109]. Instead, hypertrophic chondrocytes are characterized by the expression of type X collagen [110]. Among catabolic factors, MMP13 is expressed at a low level in normal and early degenerative cartilage, but it is strongly upregulated during inflammation [111] and contributes to cartilage degeneration via Col2a1 degradation [75,112]. Derfoul et al. [39] demonstrated that GlcN promotes chondrogenesis in mesenchymal stem cells, enhancing the expression of collagen II and reducing cartilage matrix degradation via the inhibition of MMP13 expression. In our study, under proliferative conditions, DCF001 reversed the TNFα-induced expression of MMP13 and boosted the gene expression of COL2A1 and RUNX2, thus suggesting that it is potentially able to contrast the chronic breakdown of the ECM in vivo but is unable to promote terminal chondrogenic differentiation without chondrogenic inducers. In contrast, due to promoted differentiation, DCF001 reversed the cellular response driven by TNFα, increasing the chondrogenic capacity of CMCs, as suggested by the enhanced expression of RUNX2 and MMP13 genes and the modulated transcription of COL2A1.

## 4. Materials and Methods

### 4.1. In Vitro Model

As previously published [113], multipotent stem cells were isolated from the human peripheral blood of healthy volunteer donors (*n* = 20; age: ≤12 years) in accordance with the Italian ethics committee’s authorization and after obtaining informed consent. Briefly, blood samples (5 mL) were diluted (1:1) in an α-Minimum Essential Medium (αMEM; Invitrogen Life Technologies, Carlsbad, CA, USA) and carefully layered onto Ficoll^®^Paque (Sigma-Aldrich Corp. St. Louis, MO, USA) for density gradient separation. After centrifugation (400× *g*, for 20 min, at 4 °C), the upper layer was collected and resuspended in a proliferative medium (PM) prepared with αMEM supplemented with 16.5% heat-inactivated fetal bovine serum (FBS; Invitrogen Life Technologies), 50 U/mL penicillin (Invitrogen Life Technologies), 50 μg/mL streptomycin (Invitrogen Life Technologies), and 1% L-glutamine (Sigma-Aldrich). Samples were seeded onto tissue culture plates (BD Falcon, Milan, Italy) and allowed to adhere for 24 h in a humidified atmosphere of 5% CO_2_ at 37 °C. When fibroblast colony-forming cells were observed, the culture medium was changed, and cell expansion was performed. Upon reaching 80% confluence, CMCs were detached using 0.02% ethylenediaminetetraacetic acid (EDTA)/0.25% trypsin solution (Sigma-Aldrich), and subcultures (10^4^ cells/cm^2^) were prepared for immunophenotyping and test analysis.

### 4.2. Immunophenotyping

CMCs were immunophenotypically characterized by flow cytometry (FCM). The expression of stem cell markers CD90, CD44, CD105, and CD73 and purinergic receptor CD39 was determined using the antibodies reported in Table 1. In parallel, the expression of hematopoietic marker CD45 and histocompatibility antigen HLA-DR was also evaluated (Table 1). For the analysis, cells were detached using a trypsin/EDTA solution, centrifuged at 1200 rpm for 5 min, and then resuspended in phosphate-buffered saline (PBS) (Merck, Darmstadt, Germany) and 0.2% bovine serum albumin (BSA) (Merck) (PBS-BSA). All samples were fixed using BD Cytofix™ solution (BD Biosciences, San Josè, CA, USA) following the manufacturer’s instructions. For staining, 1  ×  10^6^ cells were incubated in 100 μL of PBS and 10 μL of fluorochrome-conjugated primary antibodies (Table 1) in the dark for 15 min at room temperature (RT). In parallel, isotype-matched controls (Table 1) and unstained cells were included as negative controls. Data were acquired using a BD FACSCanto™ II System (BD Biosciences) and FACSDiva™ software v6.1.3 (BD Biosciences). Data were processed using FlowJo™ v10.8.1 Software (Tree Star Inc., Ashland, OR, USA).

### 4.3. DCF001 Compound

Tetra-acetyl GlcN hydrochloride (Sigma-Aldrich) was functionalized with tryptophan according to the previously reported procedure [114]. Briefly, hydrochloride salt, N-Cbz tryptophan (Sigma-Aldrich), diisopropylethylamine, and TBTU were suspended in dry DMF/dichloromethane 1:2 and stirred under an inert atmosphere at room temperature overnight. HCl 1 N was added, and the mixture was extracted using dichloromethane; the organic layers were dried, filtered, and evaporated under a vacuum, obtaining a white solid that underwent a deprotection reaction by adding MeONa to a suspension in methanol. After 30 min, H2SO4 was added, and the crude was evaporated under reduced pressure and washed with boiling methanol, obtaining a yellow solid as a pure compound (DCF001, molecular weight: 499.52). DCF001 was prepared in a culture medium at a final 1 µg/mL concentration.

### 4.4. Cell Treatments

CMCs were seeded (1 × 10^5^ cells/cm^2^) on standard tissue culture plates (BD Falcon). After 24 h, cells were pretreated with TNFα (0.1 µg/mL) (ImmunoTools, Friesoythe, Germany) in a PM medium for 3 h. Thus, they were cultured with DCF001 (1 µg/mL) or the vehicle (control) for 24 h in PM or a chondrogenic medium (CM) prepared with DMEM and Ham’s F12 Nutrient Mixture (Sigma Aldrich), 10% fetal calf serum (Biochrom-Seromed, Berlin/Heidelberg, Germany), 0.4 µg/mL of hydrocortisone, 8 ng/mL of cholera toxin, 5 µg/mL of insulin, 20 µg/mL of adenine, 10 µg/mL of transferrin, 10 µg/mL of triiodothyronine, and 1 ng/mL of epidermal growth factor (all purchased from Sigma).

### 4.5. Cell Viability Analysis

According to the manufacturer’s specifications, viable cells were counted using a Corning^®^ Cell Counter (Corning Inc., New York, NY, USA), and the trypan blue exclusion technique was used. Data were reported as the mean value (%) ± standard deviation (SD) (*n* = 10 samples/experimental group) from three independent experiments.

### 4.6. CellTiter-Glo^®^ Assay

CellTiter-Glo^®^ Luminescent Cell Viability Assay (Promega, Madison, WI, USA) was used and adapted to measure the ATPs released by unstimulated or stimulated cells with DCF001 and/or TNFα. Briefly, 50 µL of the culture medium of each sample was incubated with an equal volume of CellTiter-Glo^®^ Reagent in the 96-well opaque-walled plate. Luminescence was recorded 10 min after reagent addition using a VICTOR^®^ Nivo™ Plate Reader (PerkinElmer, Waltham, MA, USA) and reported in relative light units (RLU). Data were expressed as the mean ± SD of four replicates for each sample. In parallel, wells containing medium unconditioned by cells were used to measure background luminescence.

### 4.7. Flow Cytometry Analysis of CD39, CD73, and TNFα Receptors and IκBα

The analysis was performed by direct or indirect staining using the antibodies reported in Table 1. Cultures stained with isotypic, or secondary antibodies were used as references. Data were acquired using the BD FACSCanto™ II System and are expressed as follows: (1) the relative MFI values (Rel MFI) (target MFI/Isotype IgG MFI) ± standard error of the mean (SEM) for CD39 and CD73; (2) the mean value of the percentage of positive cells ± SEM for TNFR1 and TNFR2. As for the analysis of IκBα, we reported the percentage expression of phosphorylated (p-IκBα) and unphosphorylated (IκBα) proteins and the p-IκB to IκB ratio. Assuming that a ratio equal to 1 is observed in the case of undetectable differences between p-IκBα and IκBα, values greater or less than 1 were considered indicative of an increased or decreased expression of p-IκBα. The analysis was performed by flow cytometry as this technique enables the quantitative analysis of signaling events with greater sensitivity and precision than a Western blot, which is traditionally used for the analysis of protein expression but is limited to performing a multiparameter analysis of subpopulations with specific signaling responses.

### 4.8. Gene Expression Study

Total RNA was isolated from the experimental groups and evaluated by quantitative real-time PCR for the relative expression of COL2A1, RUNX2, and MMP13 mRNAs. Briefly, RNA was prepared with a TRI Reagent solution (Zymo Research, Irvine, CA, USA) following the phenol–chloroform method. After the quantification of RNA by NANODROP 2000 (Thermo Fisher Scientific, Waltham, MA, USA), the samples were stored at −20 °C until use. For the analysis, each reaction was performed with 10 ng of RNA, a one-step RT-PCR kit (qPCR SyGreen 1-step Go Lo Rox, PCR Biosystems, UK), and Mic qPCR Cycler (Bio Molecular Systems, Australia). Oligonucleotides for target genes and housekeeping hypoxanthine–guanine phosphoribosyltransferase (HPRT) (listed in Table 2) were purchased from Invitrogen Life Technology (Carlsbad, CA, USA). The relative gene expression level was measured using the Livak and Schmittgen method [115], which is also referred to as the 2^−ΔΔCt^ method [116]. Each expression was calculated relative to human HPRT and control samples in the growth medium.

### 4.9. Statistical Analysis

Each experiment was performed three times. Statistical significance was established using a two-way analysis of variance (ANOVA) followed by Bonferroni’s multiple comparison test. In all analyses, a *p*-value of <0.05 was considered statistically significant.

## 5. Conclusions

GlcN salts constitute a new class of nutraceutical components with putative chondroprotective activity. In our study, DCF001 demonstrably preserved cell viability and the chondrogenic potentialities of circulating multipotent stem cells, suggesting significant potentialities for in vivo targeting endogenous MSCs that are mobilized to damage sites and participate in cartilage regeneration or repair. Further in vivo studies are necessary to validate the observed chondroprotective effects of DCF001 during inflammation and to provide a rational basis for the development of innovative therapies for degenerative cartilage diseases.

## Figures and Tables

**Table 1:** Antibodies used for flow cytometry analysis.

**Primary Antibodies**	**Manufacturing Company**
Mouse anti-human CD39	Santa Cruz Biotecnology, Inc. (Dallas, TX, USA)
FITC mouse anti-human CD44	BioLegend, Inc. (San Diego, CA, USA)
PE mouse anti-human CD45	Santa Cruz Biotecnology, Inc.
PE mouse anti-human CD73	BioLegend, Inc.
FITC mouse anti-human CD90	Santa Cruz Biotecnology, Inc.
PE mouse anti-human CD105	Santa Cruz Biotecnology, Inc.
PE mouse anti-human HLA DR	Santa Cruz Biotecnology, Inc.
Rabbit anti-human TNFRI	Immunological Sciences (Rome, Italy)
Rabbit anti-human TNFRII	Immunological Sciences
Mouse anti-human phospho-IκBα (Ser32/36)	Cell Signaling Technology (Danvers, MA, USA)
Rabbit anti-human IκBα	Cell Signaling Technology
**Isotype controls**	**Manufacturing Company**
FITC Isotype Control	Santa Cruz Biotechnology, Inc.
PE Isotype Control	BD Biosciences (San Jose, CA, USA)
**Secondary antibodies**	**Manufacturing Company**
Alexa Fluor^®^ 488-conjugated anti-rabbit secondary antibody	Invitrogen (Waltham, MA, USA)
Alexa Fluor^®^ 488-conjugated anti-mouse secondary antibody	Invitrogen

**Table 2:** Oligonucleotides used for RT qPCR analysis.

Target Gene	Acronym	Sequence (5′–3′)	Reference Sequence
Hypoxanthine Phosphoribosyltransferase1	HPRT1	F: TGGACAGGACTGAACGTCTTGCT R: TTGAGCACACAGAGGGCTACAATG	NM_000194.2
Collagen type II alpha 1 chain	COL2A1	F: CGGGCAGAGGGCAATAGCAGGTT R: CAATGATGGGGAGGCGTGAG	NM_001844.4
Runt-related transcription factor	RUNX2	F: TCCGGAATGCCTCTTGCTGTTATGA R: ACTGAGGCGGTCAGAGAACAAACT	BC108919
Matrix metallopeptidase 13	MMP13	F: GTTGGTCCGATGTAACTCCTC R: GAAGTCGCCATGCTCCTTAAT	NM_002427
